# Multi frame holograms batched optimization for binary phase spatial light modulators

**DOI:** 10.1038/s41598-024-70428-0

**Published:** 2024-08-21

**Authors:** Jinze Sha, Antoni Wojcik, Benjamin Wetherfield, Jianghan Yu, Timothy D. Wilkinson

**Affiliations:** 1 Department of Engineering, University of Cambridge, Cambridge, CB3 0FA UK; 2 Department of Architecture, University of Cambridge, Cambridge, CB2 1PX UK

**Keywords:** Computer-generated holography, Phase retrieval, Multi-frame hologram, Optical techniques, Adaptive optics, Displays, Electrical and electronic engineering

## Abstract

Phase retrieval methods used in computer generated holograms such as Gerchberg-Saxton and gradient descent give results which are prone to noise and other defects. This work builds up on the idea of time-averaging multiple hologram frames, first introduced in methods like One-Step Phase-Retrieval and Adaptive One-Step Phase-Retrieval. The proposed technique called Multi-Frame Holograms Batched Optimization uses the L-BFGS optimization algorithm to simultaneously generate a batch of binary phase holograms which result in an average reconstructed image of improved fidelity and fast algorithmic convergence, both in the Fraunhoffer and the Fresnel regimes. The results are compared to One-Step Phase-Retrieval and Adaptive One-Step Phase-Retrieval in simulation and experimentally, proving the superiority of the proposed approach. This technique can be easily extended to other spatial modulation methods.

## Introduction

Computer-generated hologram (CGH) enables producing three-dimensional (3D) multi-depth image reconstruction via modulating the wavefront of a coherent light source. Currently available spatial light modulators (SLM) can only modulate either phase or amplitude, so algorithms are needed to compute amplitude-only or phase-only holograms. The classic phase-retrieval algorithms include direct binary search^1^, simulated annealing^2^ and Gerchberg-Saxton^3^. With the developments in modern numerical optimization methods and increase in computational power, phase retrieval with new numerical optimization methods has also been found in the literature such as: gradient descent^4,5^, its stochastic variations^6–8^, and its derivative L-BFGS^9,10^. However, all of these are single-frame hologram generation methods. In contrast, time multiplexing multi-frame holograms seeks to improve a time-averaged response by displaying different hologram sub-frames at a high refresh rate^11^. Such approach can exploit the finite response time of human vision, where human eyes average out the unwanted noise while the wanted signal remains. Similarly, such method could be used in holographic systems which require high precision without any hard restrictions on the projection refresh rate, such as holographic photo-lithography^12^. A few time-multiplexed multi-frame holograms generation methods have been explored in the literature, including the One-Step Phase-Retrieval (OSPR) algorithm^13^ and the Adaptive One-Step Phase-Retrieval (AD-OSPR) algorithm^14^; however, both OSPR and AD-OSPR are still subject to defects in reconstruction quality.

This paper therefore extends on the previous research using the L-BFGS optimization algorithm for single-frame phase-only hologram generation^9,10^, and proposes a novel time-multiplexed multi-frame holograms generation method using L-BFGS optimization, called Multi-Frame Holograms Batched Optimization (MFHBO), to produce better reconstruction quality than the existing OSPR and AD-OSPR methods.

## Method

This paper proposes a novel method of using numerical optimization algorithm L-BFGS^15^ to generate multi-frame binary-phase holograms. The L-BFGS algorithm had previously been used for single-frame hologram optimization^9,10^. To implement it onto multi-frame holograms generation, the argument to vary becomes the set of holograms with *n* sub-frames ($\{{{\textbf {H}}}_1, {{\textbf {H}}}_2,\ldots , {{\textbf {H}}}_n\}$), each having a resolution of $X\times Y$ pixels matching the resolution of the target image, and the objective function to minimise is therefore the difference between the average reconstruction amplitude $\left( {{\textbf {R}}}_{avg}=\frac{1}{n}\sum _{i=1}^n{{\textbf {R}}}_i\right)$ and the target image (${{\textbf {T}}}$), which is denoted as $Loss({{\textbf {T}}}, {{\textbf {R}}}_{avg})$, where *n* is the total number of frames, ${{\textbf {R}}}_i$’s are reconstructions from individual hologram sub-frames ${{\textbf {H}}}_i$’s for $i\in [1,n]$. To compute each ${{\textbf {R}}}_i$ from the corresponding ${{\textbf {H}}}_i$, we start from the Fresnel diffraction formula given in Eq. (1)^16^

1 $$\begin{aligned} {{\textbf {E}}}_{Fresnel\ region}(\alpha , \beta , z) = {\mathcal {F}} \left\{ {{\textbf {A}}}(x,y) \cdot e^{j\frac{k}{2z}(x^2+y^2)}\right\} \end{aligned}$$

where ${{\textbf {E}}}$ is the reconstructed electric field, in complex form, ${{\textbf {A}}}$ is the hologram aperture, also in complex form, and ${\mathcal {F}}$ denotes the Fourier Transform, implemented on computers using the Fast Fourier Transform (FFT) function. As eyes cannot perceive phase, the reconstruction amplitude is therefore the absolute value, giving ${{\textbf {R}}} = \left|{{\textbf {E}}} \right|$. And as we are generating holograms for phase-only SLM’s, ${{\textbf {A}}}$ is then comprised of a uniform amplitude with phase **H**, giving ${{\textbf {A}}} = e^{j{{\textbf {H}}}}$, where the exponential is taken element-wise.

**Figure 1:**
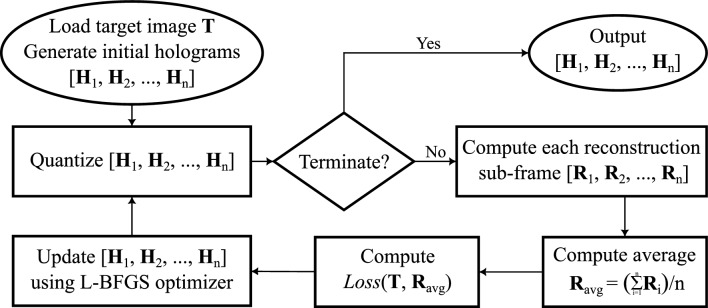
MFHBO flowchart.

To help explain the optimization process, a flow chart is drawn in Fig. 1. As shown in the flowchart, the target image ${{\textbf {T}}}$ is first loaded, with a set of *n* hologram sub-frames ($\{{{\textbf {H}}}_1, {{\textbf {H}}}_2,\ldots , {{\textbf {H}}}_n\}$) generated randomly. Then at every iteration, each hologram sub-frame ${{\textbf {H}}}_i$ is quantized to the bit-depth constraint of the SLM, and propagated to the reconstruction plane ${{\textbf {R}}}_i$, and the average of the amplitudes of all reconstructions ${{\textbf {R}}}_{avg}$ is computed and compared against the target image ${{\textbf {T}}}$ using a loss function $Loss({{\textbf {T}}}, {{\textbf {R}}}_{avg})$, after which the search direction is computed using the L-BFGS optimizer and the hologram sub-frames are updated accordingly. Here the loss function selected is the relative entropy^17^ given in Eq. (2).

2 $$\begin{aligned} Loss({{\textbf {T}}}, {{\textbf {R}}}_{avg}) = -\sum _{x=1}^{X} \sum _{y=1}^{Y} {{\textbf {T}}}_{(x,y)}\log \left( \frac{{{\textbf {R}}}_{avg(x,y)}}{{{\textbf {T}}}_{(x,y)}}\right) \end{aligned}$$

Since fast SLM’s available in the lab are binary-phase devices, the quantization step in the flowchart in Fig. 1 is carried out with bit-depth limit of 1, hence producing binary-phase holograms. However, the optimization algorithm does not converge with a straight binary quantization as integers are discrete, therefore a Sigmoid function^18^ is used for a smoother and differentiable quantization, as defined in Eq. (3). The output of the Sigmoid function is then scaled by $\pi$ so that the binary phase levels are 0 and $\pi$.

3 $$\begin{aligned} \textrm{sigmoid}(x)=\frac{1}{1+e^{-x}} \end{aligned}$$

And finally, when displaying the multi-frame holograms, each of the *n* frames generated are then rounded to binary phase values and displayed on the binary phase SLM sequentially. And when the first round finished, the second round starts with the first frame again (i.e. after frame *n*, the next frame displayed is frame 1), and such infinite loop doesn’t stop until another set of holograms are uploaded.

## Results

### Simulation results

**Figure 2:**
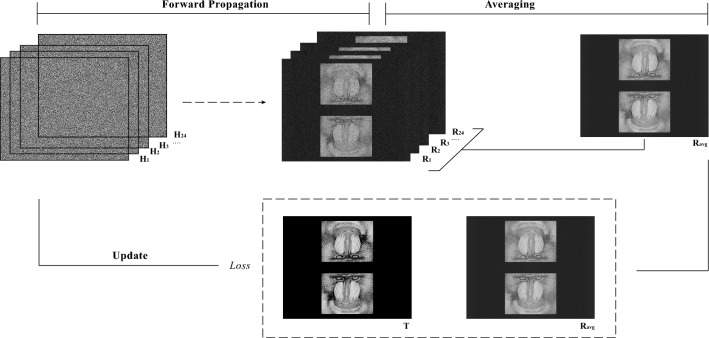
An example iteration in the optimization process.

To test the proposed MFHBO method, a target image ${{\textbf {T}}}$ as shown in Fig. 2 was used. It was designed from the widely used mandrill image^19^. A rotational symmetry was introduced to match the rotational symmetric property of the far field projections from binary phase holograms. It was then zero padded to a resolution of $1024 \; px\times 1024 \; px$ and subsequently interpolated to a resolution of $1280 \; px\times 1024 \; px$ to match the resolution of the SLM in our lab. Note that the target image was zero padded to a square aspect ratio and then stretched to the non-square aspect ratio because more pixels in the horizontal axis only means higher sampling rate as part of the features of the FFT, the replay field is continuous and is not pixelated and the simulated reconstruction of $1280 \; px\times 1024 \; px$ resolution is the sampled results, which will be illustrated visually in Fig. 5 later.

**Figure 3:**
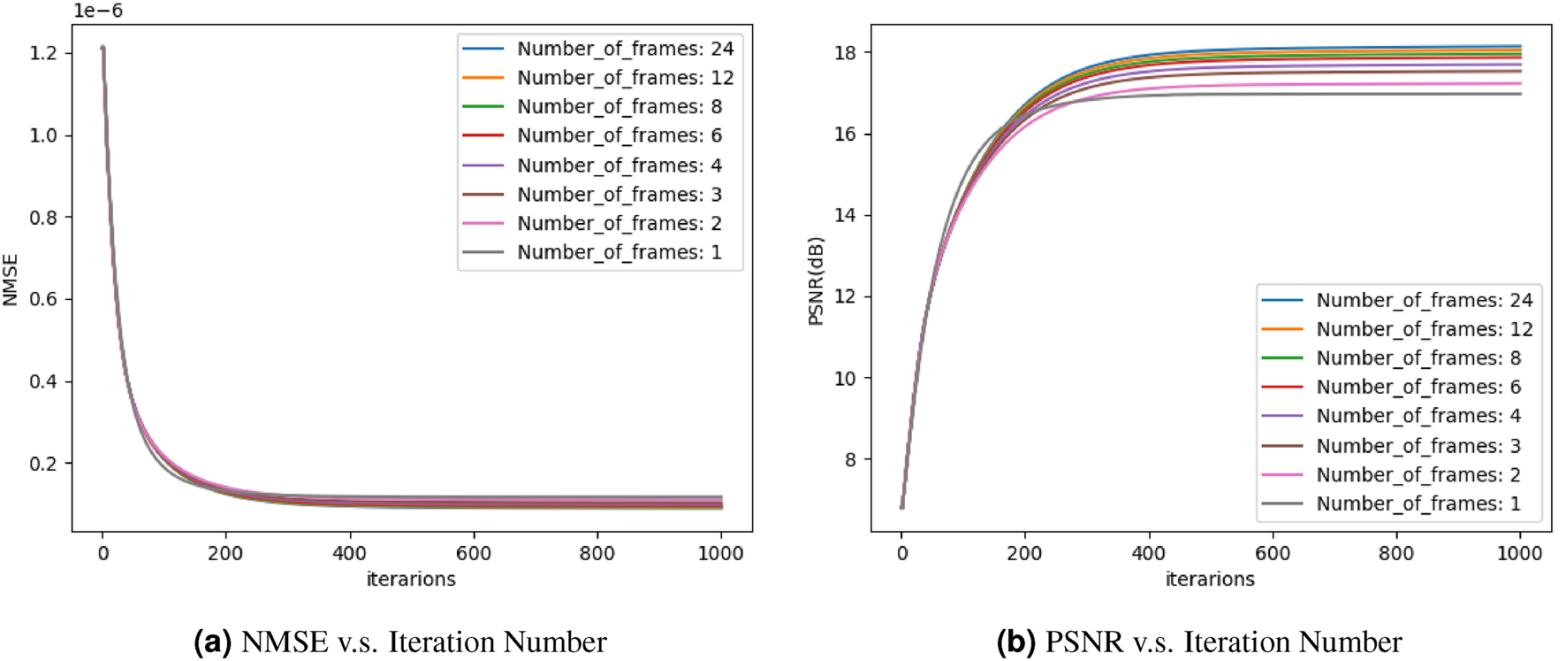
Convergence of optimization.

To further explain the optimization process described in Fig. 1, an example iteration with $n=24$ is shown in Fig. 2. At each iteration, every hologram is quantized and propagated to the reconstruction plane, forming $\{{{\textbf {R}}}_1, {{\textbf {R}}}_2,\ldots , {{\textbf {R}}}_{24}\}$. The average reconstruction amplitude ${{\textbf {R}}}_{avg}$ is then compared against the target image ${{\textbf {T}}}$, using the loss function in Eq. (2). The holograms $\{{{\textbf {H}}}_1, {{\textbf {H}}}_2,\ldots , {{\textbf {H}}}_{24}\}$ are then updated according to the search direction calculated using the L-BFGS optimizer. After setting the optimization to terminate when the number of iterations reach 1000, the same algorithm was run on the same target for different number of frames (*n*), the normalised mean squared error (NMSE) and the peak signal-to-noise ratio (PSNR) between the average reconstructions ${{\textbf {R}}}_{avg}$ and the target image ${{\textbf {T}}}$ were calculated at every iteration and plotted in Fig. 3a,b respectively.

The plots in Fig. 3 show that the proposed MFHBO method has achieved good convergence within 400 iterations, for the various number of frame settings *n* in $\{1, 2, 3, 4, 6, 8, 12, 24\}$. The final NMSE values in Fig. 3a are difficult to distinguish in the plot, therefore it will be further compared in the bar chart in Fig. 5. The number of frames are chosen to be integer factors of 24, which is determined by our experimental setup, further explained in the next subsection.

**Table 1:** MFHBO runtime (s).

Number of frames	200 iterations	400 iterations	600 iterations	800 iterations	1000 iterations
1	1.79	3.59	5.31	7.00	8.72
2	2.82	5.59	8.34	11.11	13.88
3	3.84	7.67	11.45	15.21	19.00
4	4.93	9.83	14.70	19.58	24.47
6	6.95	13.87	20.76	27.58	34.50
8	8.87	17.67	26.54	35.60	44.56
12	12.95	25.79	38.63	51.47	64.30
24	51.81	101.43	151.09	201.08	251.15

The programme runtime of the proposed MFHBO method has been measured on a laptop computer of model ASUS ROG Zephyrus M16 (GU603H) with a CPU of model i7-11800H and a GPU of model NVIDIA RTX3060 and the results for different combinations of number of frames and number of iterations are listed in Table 1. It can be concluded that the application of the proposed method is for pre-computed high-quality holograms, instead of real-time holographic projections.

### Optical experiment results

**Figure 4:**
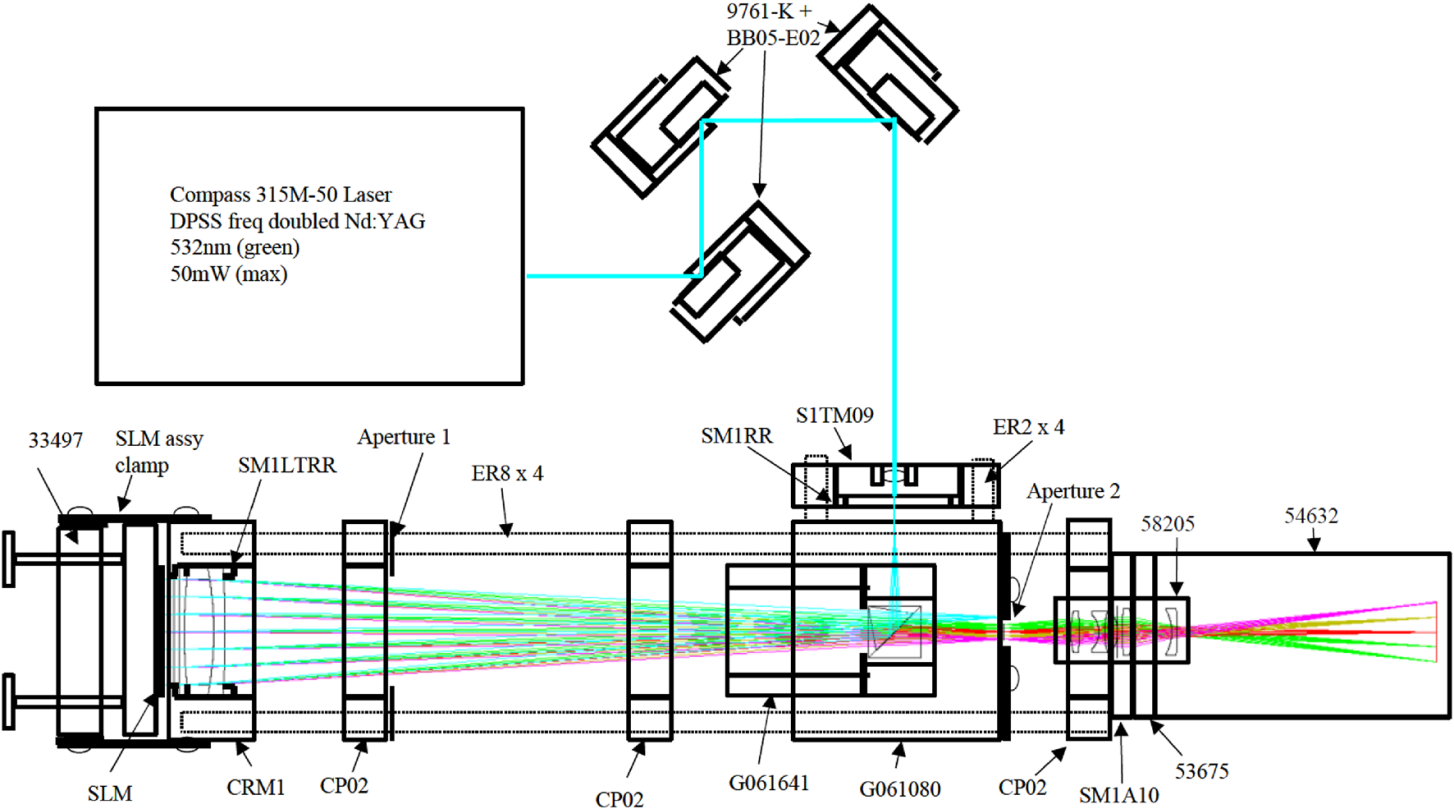
Holographic projection system components^20^.

The holographic projection system used in this experiment is the same as the one used in previous research^21^, which was originally developed by Freeman^20^. The optical setup is shown in Fig. 4. The design is consisted of a diode-pumped solid-state (DPSS) 532 nm 50 mW laser source, focused down by an aspheric singlet, and passed through a polarising beam splitter cube to a collimating lens, which illuminates the SLM^20^. The SLM is a binary phase SXGA-R2 ForthDD ferroelectric Liquid crystal on silicon (LCOS) micro-display with a refresh rate of 1440 Hz, a pixel pitch of 13.6 μm and a resolution of $1280\times 1024$^20^. Since the SLM has a refresh rate of 1440 Hz and modern computer monitors have refresh rate of at least 60 Hz, the maximum number of frames was chosen to be $1440/60=24$, so that each set of 24 frames will take a total of 1/60 s to display, therefore giving an equivalent refresh rate of 60 Hz. Then the integer factors of 24 were chosen so that the equivalent refresh rate becomes integer multiples of 60 Hz. The number of frames starts from 1 to help illustrate how the increase in number of frames positively affect the reconstruction quality.

**Figure 5:**
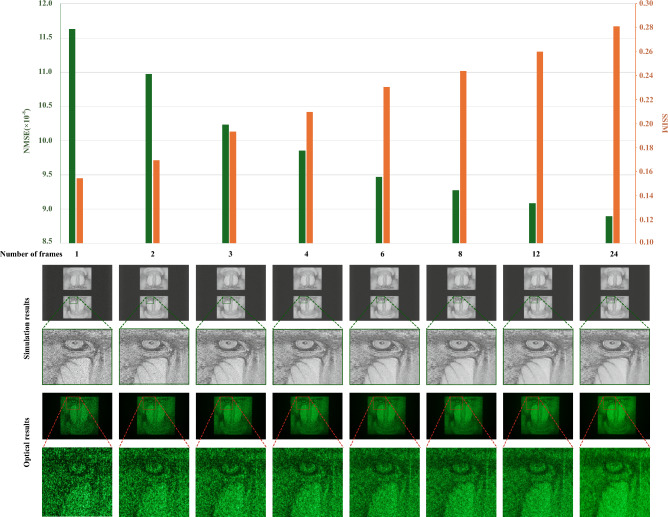
Simulation and optical reconstruction results for different number of frames.

The results in Fig. 5 further compares the final results for different number of frames. The histogram in Fig. 5 shows that, as the number of frames increases, the NMSE between the average reconstructions ${{\textbf {R}}}_{avg}$ and the target image ${{\textbf {T}}}$ decreases and the structural similarity index (SSIM)^22^ increases, showing a trend of better reconstruction quality with higher number of frames. Such trend is expected as more frames provide higher information capacity, which agrees with the previous research where holograms with higher bit depth were found to achieve better reconstruction quality^23^. The trend is also shown visually via the simulation results and their detail enlargements. The corresponding multi-frame holograms are then loaded onto the SLM, and the reconstructed field is captured using a camera of model Cannon EOS 1000D. Only the bottom halves of the reconstructed field were captured as the symmetrical conjugates were unwanted feature of far field projections from binary-phase SLM’s. The raw data including multi-frame binary-phase holograms, simulated reconstructions and optical results captured are accessible in the database^24^.

**Figure 6:**
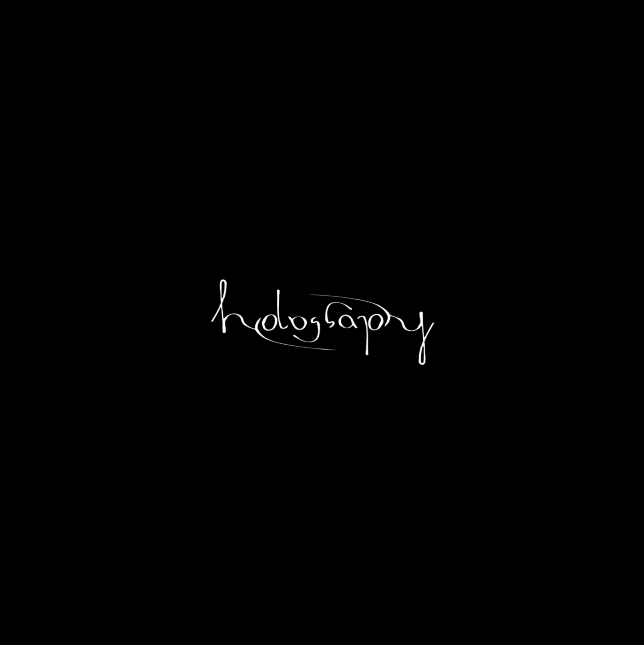
Sample target image - ‘holography’ ambigram.

**Figure 7:**
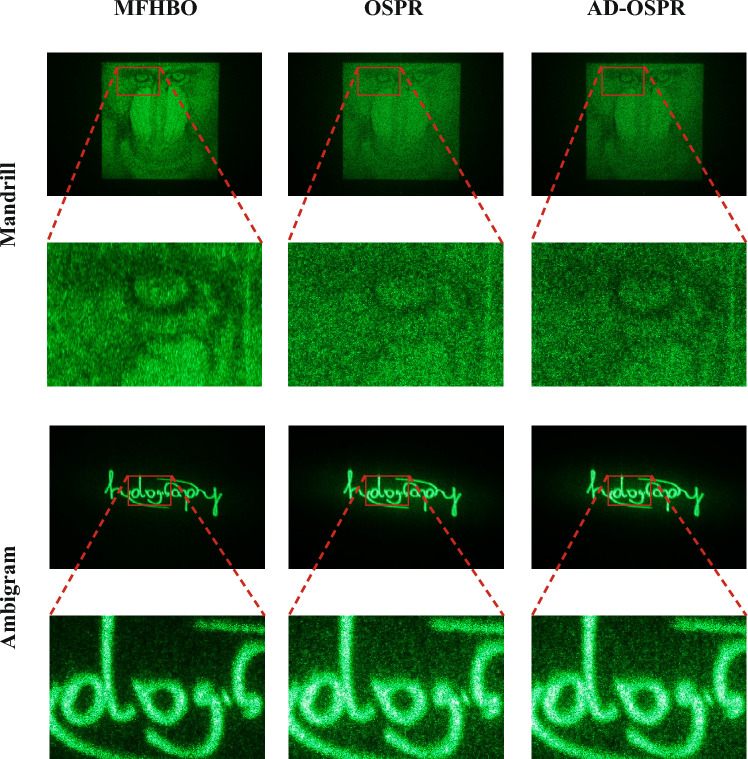
Optical results comparison of the proposed MFHBO method against the existing OSPR and AD-OSPR methods.

Then another target image was tested, which is the holography ambigram shown as shown in Fig. 6 (Adapted, with colours reversed, from *holography* - Benjamin Wetherfield, 2022). The term ambigram is used to refer to (often typographical) designs that are invariant under a reflection, rotation or other symmetry. The ‘holography’ design contains 180° rotational symmetry, which makes it especially well suited to binary Fourier-holographic projection, where this symmetry is unavoidable. Multi-frame holograms were then generated using the proposed MFHBO method and the existing OSPR and AD-OSPR methods, for the same number of frames $n=24$. And the optical results are shown in Fig. 7.

As shown in Fig. 7, for the Mandrill target image, it can be seen that the proposed MFHBO method achieved a much better optical reconstruction quality than the existing OSPR and AD-OSPR methods, with clearer details and better contrasts; for the ‘holography’ ambigram target image, the proposed MFHBO method is shown to have a much lower background noise around the centre, than the existing OSPR and AD-OSPR methods. The intended black regions are represented much more cleanly, with an elimination of speckle-like artefacts in the zero-valued space around the lettering, and an overall increase in discernible contrast.

**Table 2:** Quantitative analysis of the optical results in Fig. 7.

Image	Metric	MFHO	OSPR	AD-OSPR
Mandrill	NMSE ($\times 10^{-4}$)	0.84	1.00	1.12
	SSIM	0.124	0.076	0.078
Ambigram	NMSE ($\times 10^{-5}$)	2.29	3.31	3.23
	SSIM	0.795	0.826	0.827

A quantitative analysis was then conducted on the optical results in Fig. 7, the NMSE and SSIM between the captured reconstructions and there corresponding targets are computed and listed in Table 2. The NMSE results of the proposed MFHBO method are lower than those of the existing OSPR and AD-OSPR methods, with a 25% reduction on average among both target images. On the other hand, the SSIM results have shown a 62% increase using MFHBO than OSPR and AD-OSPR for the mandrill target image, but a slight decrease of 3.7% for the ‘holography’ ambigram target image, which is negligible as it is less than 5% and the SSIM metric is not originally designed for binary-valued non-grayscale images.

#### 3D holography

**Figure 8:**
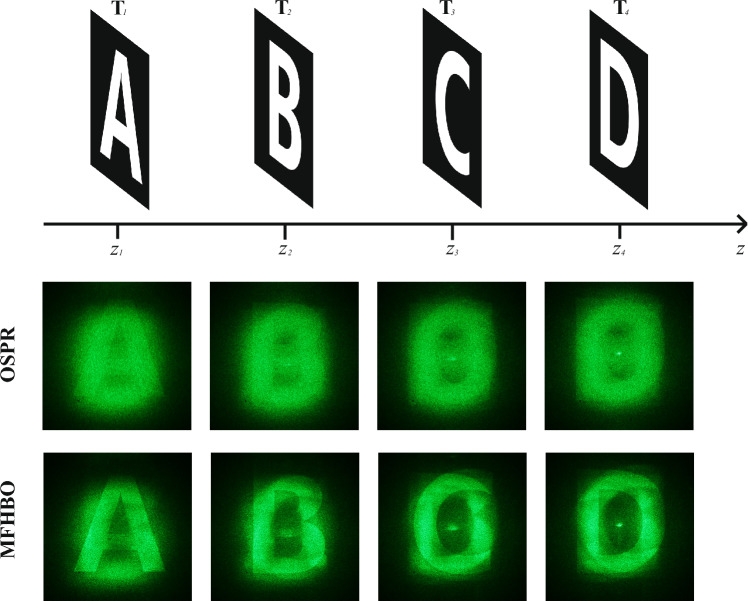
4-slice target and according reconstruction results.

The proposed MFHBO method was extended to multi-slice targets, by computing the loss between all 4 slices of reconstructions and target images (the Sum-of-Loss method in^10^). An example 4-slice target made from alphabets ‘A, B, C, D’ is shown in Fig. 8. The *z* values, corresponded to the *z* variable in Eq. (1), were chosen to be 1.1, 1.9, 3.5, 7.7 for the 4 slices respectively (as there’s no correlation between each slice, larger separation was chosen for fewer cross-talks across different planes). It can be seen that the proposed MFHBO method has produced sharper edges in reconstructions than the existing OSPR method. (The AD-OSPR method was not attempted here as its application to multi-slice targets was not defined).

**Table 3:** Quantitative analysis of the optical results in Fig. 8.

Method	Metric	Slice 1	Slice 2	Slice 3	Slice 4	Average
OSPR	NMSE ($\times 10^{-4}$)	4.980	4.484	5.644	4.846	4.988
	SSIM	0.072	0.061	0.048	0.060	0.060
MFHO	NMSE ($\times 10^{-4}$)	4.484	4.230	4.990	4.289	4.498
	SSIM	0.063	0.067	0.058	0.072	0.065

Then a quantitative analysis was carried out, with NMSE and SSIM values measured and shown in Table 3. The proposed MFHBO method has shown a 10% reduction in NMSE and a 8% improvement in SSIM on average than the existing OSPR method, demonstrating the effectiveness of the proposed method.

**Figure 9:**
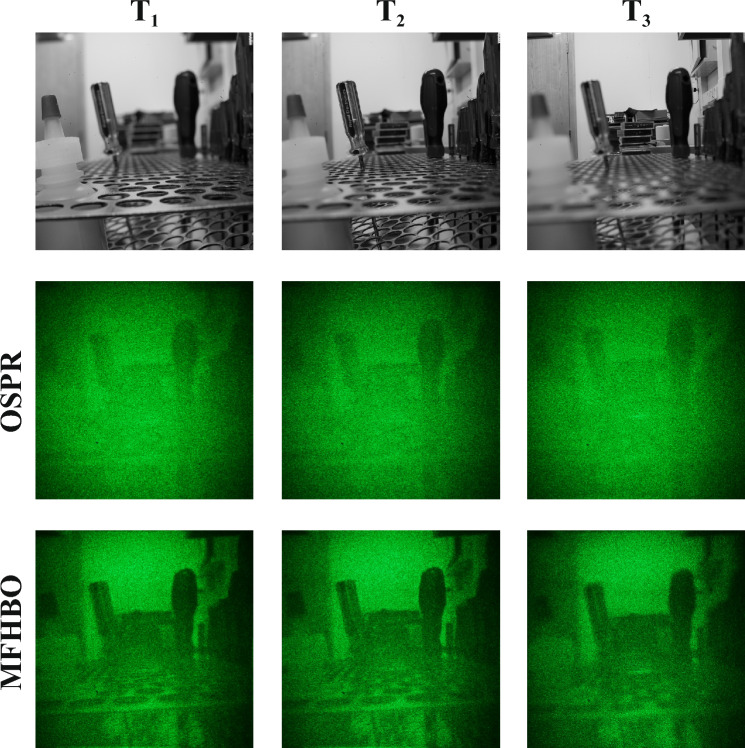
Real-life captured image as target field and their reconstruction results.

Lastly, a set of real-life scene was captured in the lab using near, middle and far focus, as shown in ${{\textbf {T}}}_1, {{\textbf {T}}}_2, {{\textbf {T}}}_3$ in Fig. 9 respectively. The *z* values were set to 1.1, 1.2, 1.3 for hologram generation, and the reconstruction results of the existing OSPR and the proposed MFHBO methods are compared in Fig. 9. The proposed MFHBO method is shown to have achieved much better reconstruction quality than the existing OSPR method.

**Table 4:** Quantitative analysis of the optical results in Fig. 9.

Method	Metric	Slice 1	Slice 2	Slice 3	Average
OSPR	NMSE ($\times 10^{-6}$)	3.70	3.69	3.47	3.62
	SSIM	0.37	0.28	0.34	0.33
MFHO	NMSE ($\times 10^{-6}$)	3.20	2.78	3.06	3.01
	SSIM	0.42	0.32	0.32	0.35

A quantitative analysis was conducted again, with NMSE and SSIM values measured and listed in Table 4. The proposed MFHBO method has shown a 17% reduction in NMSE and a 7% improvement in SSIM on average than the existing OSPR method, proving the effectiveness of the proposed method.

## Conclusion

This paper proposed the MFHBO method to generate multi-frame binary-phase holograms to be displayed on high refresh rate binary-phase SLM. The proposed MFHBO method was shown to achieve much better reconstruction quality and higher contrast than the existing multi-frame binary-phase holograms generation methods OSPR^13^ and AD-OSPR^14^ on the holographic projector with binary-phase SLM, for all the single-slice far-field targets and the multi-slice near-field targets tested. Although the propose MFHBO method is slower than the existing OSPR and AD-OSPR methods, its much better reconstruction quality makes it suitable for pre-computed high-quality hologram applications. Its strong advantage for high contrast target such as the ‘holography’ ambigram, with much suppressed speckle noise in the background, makes it well-suited for photo-lithography applications. The proposed method can also be adapted for multi-level SLM’s by simply removing the quantization step (in Fig. 1). This could be the case for applications such as photo-lithography, where the time response of the system is much longer than it is for human vision, and the high refresh rates of the SLM are not necessary.

## Data Availability

The datasets generated and/or analysed during the current study are available in the Apollo - University of Cambridge repository, 10.17863/CAM.109607.

## References

[1] Seldowitz, M. A., Allebach, J. P. & Sweeney, D. W. Synthesis of digital holograms by direct binary search. *Appl. Opt.* **26**, 2788–2798. 10.1364/ao.26.002788 (1987). 20489962 10.1364/AO.26.002788

[2] Kirkpatrick, S., Gelatt, C. D. & Vecchi, M. P. Optimization by simulated annealing. *Science* **220**, 671–680. 10.1126/science.220.4598.671 (1983). 17813860 10.1126/science.220.4598.671

[3] Gerchberg, R. W. A practical algorithm for the determination of phase from image and diffraction plane pictures. *Optik* **35**, 237–246 (1972).

[4] Zhang, J., Pégard, N., Zhong, J., Adesnik, H. & Waller, L. 3D computer-generated holography by non-convex optimization. *Optica* **4**, 1306. 10.1364/optica.4.001306 (2017).

[5] Liu, S. & Takaki, Y. Optimization of phase-only computer-generated holograms based on the gradient descent method. *Appl. Sci. (Switzerland)* **10**, 4283. 10.3390/app10124283 (2020).

[6] Chen, C. *et al.* Multi-depth hologram generation using stochastic gradient descent algorithm with complex loss function. *Opt. Express* **29**, 15089. 10.1364/oe.425077 (2021). 33985216 10.1364/OE.425077

[7] Choi, S., Kim, J., Peng, Y. & Wetzstein, G. Optimizing image quality for holographic near-eye displays with Michelson holography. *Optica* **8**, 143. 10.1364/optica.410622 (2021).

[8] Kadis, A. *et al.* Effect of bit-depth in stochastic gradient descent performance for phase-only computer-generated holography displays. *Lond. Imaging Meet.* **3**, 36–40. 10.2352/LIM.2022.1.1.09 (2022).

[9] Sha, J., Kadis, A., Yang, F. & Wilkinson, T. D. Limited-memory bfgs optimisation of phase-only computer-generated hologram for fraunhofer diffraction. In *Digital Holography and 3-D Imaging 2022*, W3A.3 (Optica Publishing Group, 2022).

[10] Sha, J., Kadis, A., Yang, F., Wang, Y. & Wilkinson, T. D. Multi-depth phase-only hologram optimization using the l-bfgs algorithm with sequential slicing. *J. Opt. Soc. Am. A* **40**, B25–B32. 10.1364/JOSAA.478430 (2023). 10.1364/JOSAA.478430 37132962

[11] Amako, J., Miura, H. & Sonehara, T. Speckle-noise reduction on kinoform reconstruction using a phase-only spatial light modulator. *Appl. Opt.* **34**, 3165–3171. 10.1364/AO.34.003165 (1995). 21052472 10.1364/AO.34.003165

[12] Bay, C., Hübner, N., Freeman, J. & Wilkinson, T. Maskless photolithography via holographic optical projection. *Opt. Lett.* **35**, 2230–2232. 10.1364/OL.35.002230 (2010). 20596203 10.1364/OL.35.002230

[13] Cable, A. J. *et al.* 53.1: Real-time binary hologram generation for high-quality video projection applications. *SID Symposium Digest of Technical Papers* vol. 35, pp. 1431 10.1889/1.1825772 (2004).

[14] Kaczorowski, A., Gordon, G. S. D. & Wilkinson, T. D. Adaptive, spatially-varying aberration correction for real-time holographic projectors. *Opt. Express* **24**, 15742–15756. 10.1364/OE.24.015742 (2016). 27410846 10.1364/OE.24.015742

[15] Liu, D. C. & Nocedal, J. On the limited memory bfgs method for large scale optimization. *Math. Program.* **45**, 503–528. 10.1007/BF01589116 (1989).

[16] Goodman, J. W. *Introduction to Fourier Optics* 4th edn. (Freeman W.H., 2017).

[17] Kullback, S. & Leibler, R. A. On information and sufficiency. *Ann. Math. Stat.* **22**, 79–86. 10.1214/aoms/1177729694 (1951).

[18] Bacaër, N. *Verhulst and the Logistic Equation (1838)* 35–39 (Springer London, 2011).

[19] Weber, A. Sipi image database - misc (2022). [Retrieved 17 Nov 2022].

[20] Freeman, J. Visor projected helmet mounted display for fast jet aviators using a fourier video projector. PhD thesis, (Department of Engineering, University of Cambridge, 2009).

[21] Sha, J., Goldney, A., Kadis, A., Skirnewskaja, J. & Wilkinson, T. D. Digital pre-distorted one-step phase retrieval algorithm for real-time hologram generation for holographic displays. *J. Imaging Sci. Technol.* **67**, 030405–1. 10.2352/J.ImagingSci.Technol.2023.67.3.030405 (2023).

[22] Wang, Z., Bovik, A., Sheikh, H. & Simoncelli, E. Image quality assessment: from error visibility to structural similarity. *IEEE Trans. Image Process.* **13**, 600–612. 10.1109/TIP.2003.819861 (2004). 15376593 10.1109/tip.2003.819861

[23] Sha, J. *et al.* Information capacity of phase-only computer-generated holograms for holographic displays. In *Optics, Photonics, and Digital Technologies for Imaging Applications VIII* (eds Schelkens, P. & Kozacki, T.) vol. 12998, p. 129980J (International Society for Optics and Photonics, SPIE, 2024) 10.1117/12.3021882.

[24] Sha, J., Wojcik, A., Wetherfield, B., Yu, J. & Wilkinson, T. D. Research data supporting “multi-frame holograms batched optimization”. Apollo - University of Cambridge Repository, 10.17863/CAM.109607 (2024).

